# Using a computerized database (REDCap) to access the clinical follow-up of patients undergoing bariatric surgery

**DOI:** 10.1016/j.clinsp.2025.100714

**Published:** 2025-06-27

**Authors:** Caroline Rossi Welendorf, Thaís Alves de Azevedo Chaves Pastore, Natália Yumi Noronha, Flávia de Campos Ferreira, Ligia Moriguchi Watanabe, Carolina Ferreira Nicoletti-Fino, Wilson Salgado Junior, Guilherme da Silva Rodrigues, Marcela Augusta de Souza Pinhel, Carla Barbosa Nonino

**Affiliations:** a Laboratory of Nutrigenomics Studies, Department of Health Sciences, Faculdade de Medicina de Ribeirão Preto, Universidade de São Paulo, Ribeirão Preto, SP, Brazil; b Applied Physiology and Nutrition Research Group, School of Physical Education and Sport, Faculdade de Medicina, Universidade de São Paulo (FMUSP), São Paulo, SP, Brazil; c Department of Anatomy and Surgery, Faculdade de Medicina de Ribeirão Preto, Universidade de São Paulo, Ribeirão Preto, SP, Brazil; d Department of Molecular Biology, Faculdade de Medicina de São José do Rio Preto, São José do Rio Preto, SP, Brazil

**Keywords:** Data collection, REDCap, Bariatric surgery

## Abstract

This study highlights the use of REDCap as an effective tool for the clinical follow-up of bariatric surgery patients. By manually entering data from medical records spanning 2000 to 2020, the study provides insights into REDCap’s capabilities in organizing and analyzing large datasets. Key findings demonstrate its value in monitoring weight changes, biochemical parameters, and adherence to supplementation protocols, contributing to improved patient management and research efficiency. While REDCap’s utility is clear, limitations include the retrospective and single-site nature of the study, which may affect generalizability. Future efforts will focus on expanding data collection and integrating the database into routine clinical workflows.

## Highlights

• Use of REDCap database for follow-up of bariatric surgery patients.
• Study shows the effectiveness of REDCap in managing bariatric surgery data.
• Electronic data platforms can optimize the management of bariatric patients.

## Methods

The present research team used REDCap® to access the clinical follow-up of patients undergoing bariatric surgery between 2000 and 2020 and manually entered from medical records. This study was approved by the Research Ethics Committee of the Ethics Committee of the Ribeirão Preto Medical School (CAAE: 44,877,521.7.0000.5440). To ensure data accuracy, all entries underwent a double-checking process. Random samples of the data were verified against source documents, and automated validation rules in REDCap flagged inconsistencies for review. Additionally, values that appeared significantly outside the expected range were further verified against the original records. Visual verification was employed as a secondary measure to identify and correct errors. This study followed the relevant guidelines for observational research, including the STROBE Statement for observational studies, ensuring methodological rigor and transparency in the data collection and analysis process.

### Setting

The audit tool and database were developed based on outpatient care protocols. The structure of the fields in the study’s database included open data fields as either text or numbers, multiple-choice fields and single-choice fields. All the data mentioned below are collected at each consultation with the multi-professional team of treatment for bariatric surgery. Usually, the patient follows a post-surgery follow-up of 10-years.

The database record consists of personal information, weight change history, smoking and alcohol consumption, presence of comorbidities, information about the surgery, anthropometry, body composition, food intake and intolerance, use of vitamin supplements and medications, number of appointments with nutritionist and psychologist, and biochemical parameters.

### Service protocol in the bariatric surgery service at hcfmrp/usp

The patient consults with the doctor preoperatively to verify evaluation techniques, including weight loss, physical activity, altered test results, and eradication of Helicobacter pylori. The nutrition and psychology team evaluates the patient's weight history, eating habits, and psychological data through interviews.

Patients are referred to groups before the surgery, where they receive multi-professional guidance about the surgery, and their psychic functioning is worked on for the changes they will face, in addition to their weight evolution being monitored and other tests are collected.

Post-surgery follow-up includes evaluation and removal of surgical stitches and drains, consultation with doctor and nutritionist to monitor diet progression, vitamin supplements, weight evolution, and biochemical tests for monitoring.

After 3-months, physical activity is allowed, and biochemical tests and ultrasounds are repeated. At 6‒12 months, follow-up consultations and assessments are conducted, including biochemical tests and bone densitometry. At 18-months, evaluations are repeated, and plastic surgery is possible if needed. The same procedures are repeated 2-years after surgery.

## Results and discussion

**Fig. 1:**
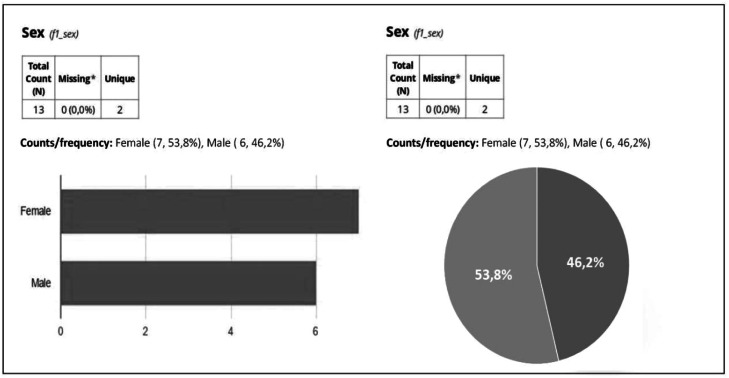
Illustration of sector charts and bar graphs with the variables gender generated by REDCap.

**Fig. 2:**
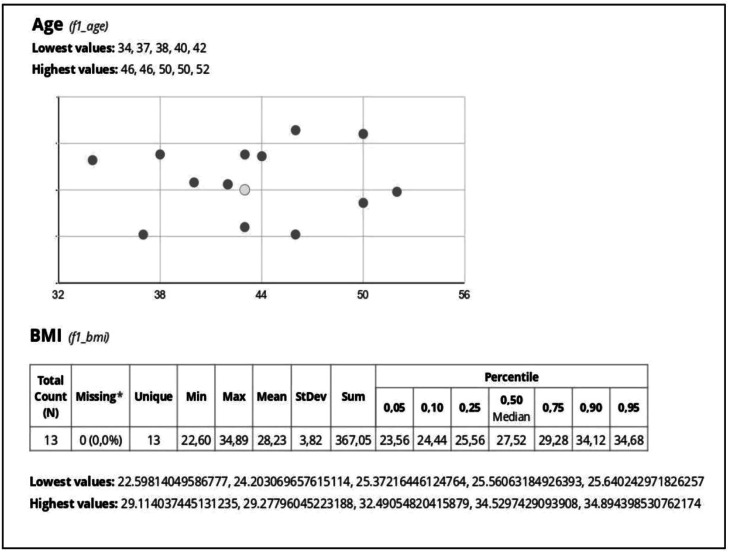
Illustration of dot plots with age and tables with BMI variables generated by REDCap.

All data entered are analyzed to carry out an evaluation of patients who underwent the surgical procedure and therefore evaluate the main complaints related to the postoperative period, weight change, evolution of biochemical tests, food consumption, adherence to vitamin supplementation, and use of alcohol and smoking. In this way, it provides results of the clinical evolution of patients over the years after surgery, for scientific purposes, with the export of data to a statistical analysis program. But it also allows easy visualization of the entire clinical history of the patient with descriptive statistics to summarize data trends, including frequencies, means, standard deviations, and percentiles. REDCap provides a preliminary analysis of frequencies and ranges, highlighting potential outliers. Fig. 1, Fig. 2 exemplify how data is generated in the form of graphs and tables, respectively, which facilitate the observation of their results, without the possibility of data loss due to the use of different users.

The data in Fig. 1 include bar charts and sector charts, such as the distribution of patients by gender, providing an overview of demographic characteristics. In contrast, Fig. 2 illustrates dot plots showing the relationship between age and BMI (Body Mass Index), as well as detailed tables that track anthropometric data variations over time. These graphical and tabular visualizations facilitate the analysis of clinical trends and support rapid and accurate data interpretation, promoting more efficient follow-up.

In REDCap, users with appropriate permissions are able to export project data and generate reports. This feature adds an additional layer of security to projects, as the main administrator assigns specific access rights to individuals.^1^ Each REDCap user is granted sole discretion in determining who can access their data. Moreover, the processing and storage of data in REDCap adhere to the Personal Data Protection Law (Law n° 13,709, August 2018), thereby ensuring the safe usage of research data.^2^

REDCap has been widely adopted in various areas of clinical research, demonstrating its versatility and effectiveness.^1^^,^^3^^,^^4^ For instance, a recent study used REDCap to monitor influenza vaccination coverage among healthcare workers, enhancing data collection and analysis, which allowed for precise evaluations of vaccination rates and targeted improvements. Additionally, REDCap was employed in a randomized clinical trial during the COVID-19 pandemic, facilitating participant recruitment, scheduling laboratory visits, and data collection while ensuring data integrity and confidentiality. These examples highlight REDCap’s adaptability in different clinical research contexts, reinforcing its utility as a robust tool for data management.

The major advantages of using this software are long-term reduction of research costs, the possibility of utilization on both smart devices and desktop computers, and rapid data entry, review, and statistical analysis.^1^^,^^3^, ^4^, ^5^, ^6^

The reliance on data from a single institution means that findings are shaped by the specific practices, patient demographics, and resources of this site, which may differ from other settings. Furthermore, as a retrospective study, the data is constrained by the accuracy and completeness of historical records, which could introduce biases. These limitations highlight the need for multicenter studies to validate and extend the applicability of these results to broader populations and diverse healthcare environments. Future research should involve multicenter data to enhance external validity.

The use of REDCap® for the collection, organization, and structured analysis of clinical data has significant implications by providing a robust and adaptable framework for data management in healthcare services that require longitudinal patient monitoring. The database design ensures that key variables are systematically collected and stored, enabling reproducible analyses and facilitating multicenter collaborations. These findings, combined with the tool’s advantages, underscore its potential to improve clinical decision-making and standardize protocols, enhancing both patient care and scientific research across various healthcare environments.

After gathering retrospective data, the authors intend to continue to enter the data of patients who underwent surgery after 2020 and extend the database collection to continue evaluating the service and also, implement its daily use in outpatient care.

## Conclusion

REDCap is an effective and cost-effective tool for research development, with ease of use in creating, data entry, and exporting data. Visual verification during data entry minimizes the risks of errors. It assists in organizing and standardizing data collection and can guide professionals in filling in necessary variables. Specifically for services that have a high demand from patients, REDCap can assist in the organization and standardization of data collection, as well as in clinical practice as an effective tool in scientific research and can help professionals completely evaluate patients during each consultation/return.

This study underscores REDCap’s role in clinical research. By standardizing data collection and offering intuitive visualization tools, it supports efficient patient follow-up and enhances data reliability. Comparable studies using REDCap for other clinical conditions corroborate its adaptability and effectiveness. Beyond bariatric surgery, the use of REDCap can be extended to various areas of clinical research, such as chronic disease management and multicenter studies. Its ability to centralize and standardize data collection promotes consistency and facilitates collaboration among institutions. Additionally, REDCap’s cost-effectiveness and scalability make it a valuable tool for resource-limited settings, potentially improving healthcare research and delivery on a broader scale.

## Ethical considerations

All procedures performed in studies involving human participants were in accordance with the ethical standards of the institutional and national research committee and with the 1964 Helsinki Declaration and its later amendments or comparable ethical standards.

## Confidentiality measures

Patient confidentiality was maintained throughout the data entry and storage process in REDCap. Protocols to protect confidential health information have been established. Restricted access to authorized research team members, data encryption, and anonymization of personal identifiers are examples. Additionally, data processing and storage complied with the Personal Data Protection Law (Law n° 13,709, of August 2018), ensuring compliance with legal and ethical standards for the processing of sensitive health data.

Informed consent was obtained from all individual participants included in the study.

## CRediT authorship contribution statement

**Caroline Rossi Welendorf:** Conceptualization, Writing – original draft, Visualization. **Thaís Alves de Azevedo Chaves Pastore:** Conceptualization, Writing – original draft, Visualization. **Natália Yumi Noronha:** Conceptualization. **Flávia de Campos Ferreira:** Writing – original draft. **Ligia Moriguchi Watanabe:** Methodology. **Carolina Ferreira Nicoletti-Fino:** Writing – review & editing. **Wilson Salgado Junior:** Supervision. **Guilherme da Silva Rodrigues:** Conceptualization. **Marcela Augusta de Souza Pinhel:** Supervision. **Carla Barbosa Nonino:** Supervision, Project administration.

## Declaration of competing interest

The authors declare no conflicts of interest.
